# Endogenous Endophthalmitis Heralding Central Nervous System Involvement by Nocardia Farcinica

**DOI:** 10.7759/cureus.9896

**Published:** 2020-08-20

**Authors:** Luis E Aguirre, Rolando A Zamora Gonzalez, Priscila Barreto-Coelho, Nicolas A Yannuzzi, Sabrina N Taldone

**Affiliations:** 1 Internal Medicine, University of Miami Miller School of Medicine/Jackson Memorial Hospital, Miami, USA; 2 Ophthalmology, University of Miami Miller School of Medicine/Bascom Palmer Eye Institute, Miami, USA

**Keywords:** nocardia, endophthalmitis, immunosuppression, brain abscess, nocardiosis, ground-glass opacity

## Abstract

Nocardiosis is an uncommon opportunistic infection caused by aerobic, gram-positive, weakly acid-fast, filamentous bacteria of the genus Nocardia that presents as a suppurative disease in immunocompromised hosts. Herein the authors describe the case of an elderly male with granulomatosis with polyangiitis (GPA) on chronic immunosuppressive therapy that presented initially with visual symptoms and developed focal neurological deficits. Nocardia should be considered as a potential pathogen in any immunosuppressed patient presenting with endogenous endophthalmitis and new-onset focal neurological deficits. Early recognition and treatment may prevent irrevocable neurological compromise stemming from misdiagnosis.

## Introduction

Nocardiosis is an uncommon opportunistic infection caused by aerobic, gram-positive, weakly acid-fast, filamentous bacteria of the genus Nocardia that presents as a suppurative disease in immunocompromised hosts [1, 2]. Central nervous system (CNS) involvement has been reported in up to 44% of cases and is seen in the context of disseminated disease [3].

## Case presentation

A 71-year-old male with a history of granulomatosis with polyangiitis (GPA) on immunosuppressive therapy and endogenous endophthalmitis of the right eye (OD) presented to the emergency room (ER) with one week of malaise, cough, worsening vision, cephalgia, altered mental status, and gait imbalance. He denied seizures.

**Figure 1:**
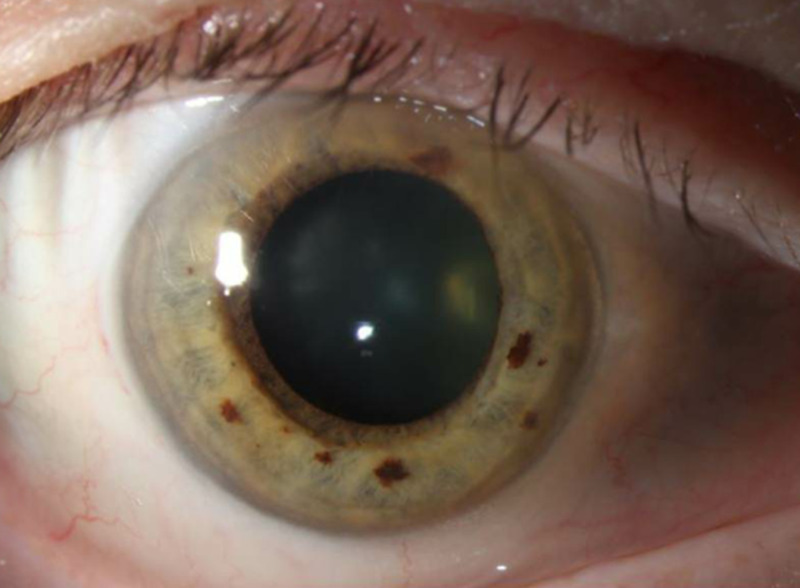
External slit-lamp photograph of the right eye External photograph of the right eye disclosing minimal anterior segment inflammation.

GPA had been diagnosed six months prior, and he had been on maintenance therapy with daily prednisone and monthly cyclophosphamide since diagnosis. Endophthalmitis had been diagnosed three months prior when the patient presented to an outside hospital with floaters and restricted OD gaze due to ocular pain. Slit-lamp examination at that time disclosed anterior segment inflammation (Figure 1).

**Figure 2:**
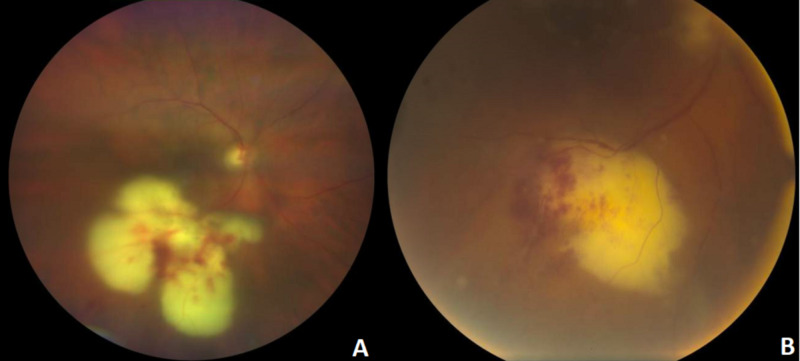
Color fundus photography of the right eye A: fundoscopy of the right eye. B: a magnified image of the lesion, disclosed a large, yellowish/white chorioretinal lesion involving the macula and inferior periphery with pseudopodal projections and associated hemorrhage.

Fundoscopy showed punctate hemorrhages and effusions in the macula of the OD with bullous lesions extending into the periphery of the eye and fibrotic opacities (Figure 2).

Vitreous cultures were negative. Features were highly suspicious of fungal etiology in the context of immunosuppression. He received intravitreal vancomycin, ceftazidime, and voriconazole and was instructed to continue systemic antifungal therapy. Clinical examination two weeks later disclosed worsening chorioretinitis; however, voriconazole was discontinued after developing intention tremor and dysdiadochokinesia raising concerns for drug-related toxicity. The patient was transitioned to oral posaconazole in view of worsening pain and vision.

**Figure 3:**
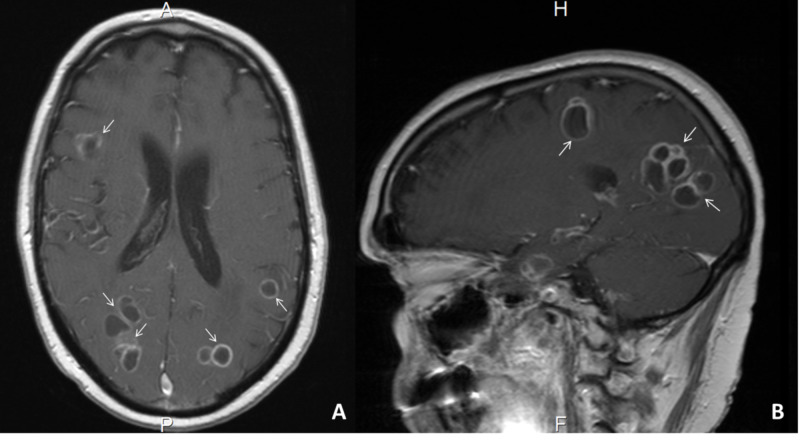
Neuroimaging studies T1-weighted MRI of the Brain (A - axial and B - sagittal views). Slices show multiple multifocal and well-circumscribed supratentorial lesions with restricted diffusion, vasogenic edema and focal mass effect with budding daughter lesions.

In the emergency room, physical examination was significant for motor ataxia, lack of coordination, dysmetria, dysdiadochokinesia, tremor, unsteady gait, and lethargic speech. Visual acuity, motility, and red-green color perception were severely compromised. Preliminary workup revealed leukocytosis of 21,000 with associated left shift. Brain MRI revealed multiple multifocal and well-circumscribed supratentorial lesions with restricted diffusion, vasogenic edema, and focal mass effect with budding daughter lesions (Figure 3). Routine electroencephalogram (EEG) was negative for epileptic discharges but revealed left frontotemporal slowing suggestive of cerebral dysfunction and moderate encephalopathy.

Findings were concerning for disseminated fungemia with metastatic seeding and CNS involvement. Transthoracic echocardiogram was negative for valvular lesions. A lumbar puncture was performed and neurosurgery was consulted for potential stereotactic drainage of abscesses. Preliminary studies of the cerebrospinal fluid (CSF) were negative including Fungitell, galactomannan, cryptococcus antigen, and Mycobacterium tuberculosis (MTB) polymerase chain reaction (PCR). Lumbar puncture showed neutrophilic pleocytosis (625 nucleated cells, 84% neutrophils) increased protein (94 mg/dl); and normal glucose levels (55 mg/dl). Gram stain showed many white blood cells but no organisms. Acid-fast bacilli (AFB) stains and fungal microscopy were also negative.

**Figure 4:**
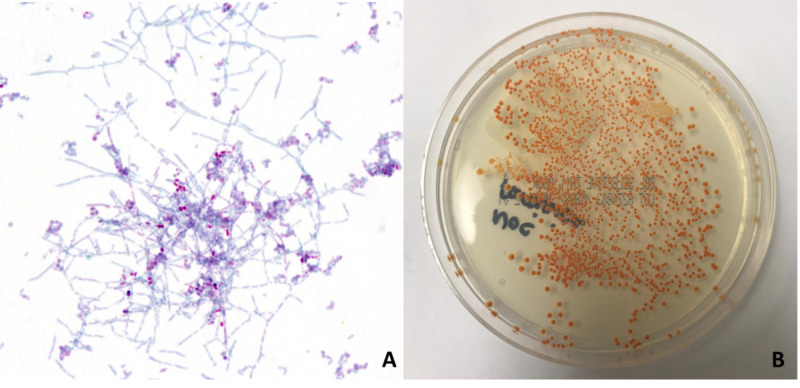
Nocardia farcinica A: gram stain (100x magnification) shows aerobic, gram-positive, weakly acid-fast, filamentous bacteria with branched vegetative hyphae. B: in vitro, Nocardia colonies have a white, orange, yellow, or brown appearance and require usually 5-21 days to grow.

Cyclophosphamide was discontinued, prednisone was tapered and the patient was started on vancomycin, meropenem, and liposomal amphotericin B. The patient’s mentation and condition improved. By the third week of hospitalization, CSF culture grew Nocardia farcinica (Figure 4).

**Figure 5:**
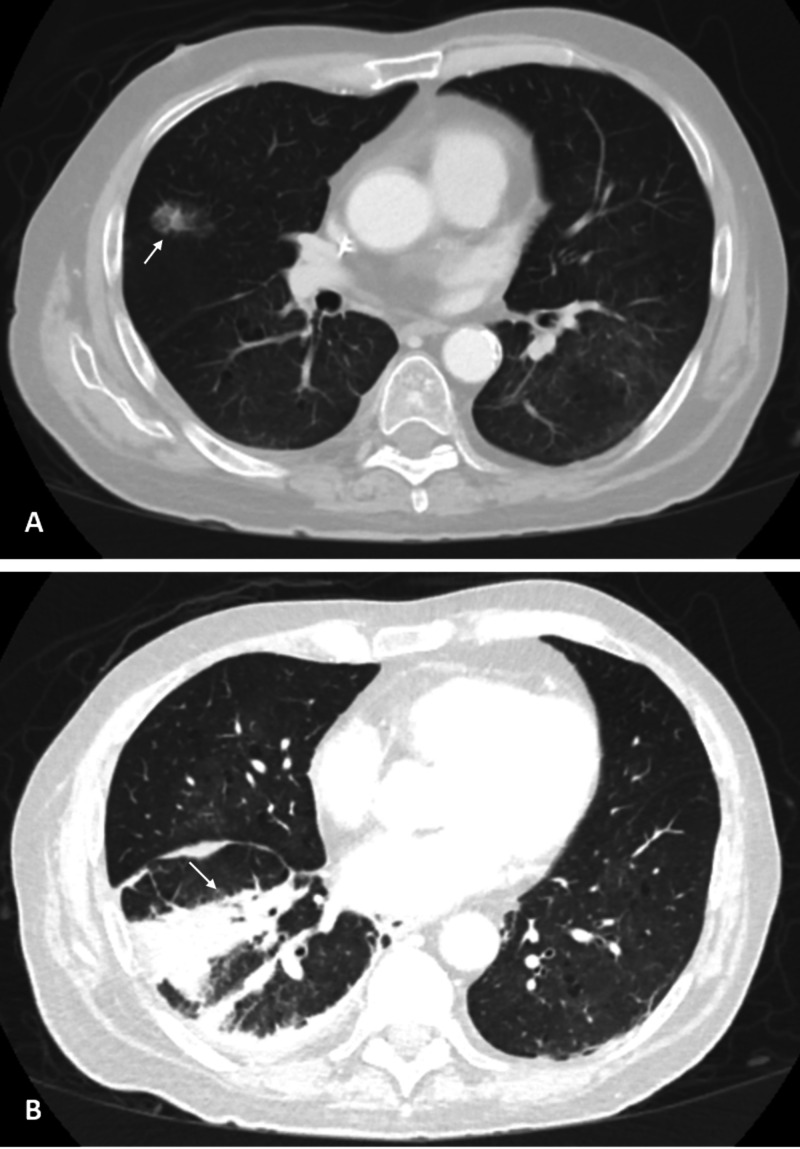
Chest imaging studies A: CT of the chest showing nodule in the right middle lobe with ground-glass and spiculated solid components. B: right lower lobe opacity with ground-glass and airspace components with a peripheral component and linear component extending centrally associated with pleural effusion/thickening.

CT imaging of the chest obtained as part of a comprehensive evaluation to investigate for a primary source of infection was significant for extensive lung involvement: there were multiple subcentimeter nodules bilaterally, a 2.9 x 1.4 cm right middle lobe pulmonary nodule with ground-glass and spiculated solid component and opacity in the right lower lung associated with pleural effusion/thickening (Figure 5). Findings were suggestive of pulmonary nocardiosis. The appearance of the patient’s lesions was consistent with disseminated nocardiosis from a primary pulmonary source causing endogenous endophthalmitis and CNS involvement, and antibiotic coverage was narrowed to trimethoprim-sulfamethoxazole (TMP-SMX) and imipenem.

Follow-up neuroimaging one month after presentation showed persistent multifocal lesions with improved edema. No drainage of abscesses was attempted by neurosurgery owing to patient preference, remarkable clinical improvement, interim decrease in size, and identification of organisms allowing for narrowing of antibiotic therapy. The patient was discharged on TMP-SMX and imipenem to complete at least six months of oral and six weeks of parenteral treatment, respectively.

## Discussion

Nocardiosis is an uncommon and primarily opportunistic infection caused by bacteria in the genus Nocardia, of which there are 13 clinically relevant species [2]. Most cases are seen in the context of immunocompromise, with up to one third being reported in immunocompetent individuals [3, 4]. The disease has a worldwide distribution, with an annual incidence of approximately 0.375 cases per 100,000 population in North America, Europe, and Australia [5].

Infection is usually acquired via inhalation of the organism into the lower respiratory tract from contaminated soil. It can present as either localized disease (most commonly affecting the lung) or with multifocal involvement caused by hematogenous dissemination from a primary focus. CNS seeding represents the main site of extrapulmonary infection, where it tends to form abscesses [2]. In our patient, the primary source of infection was thought to be the lungs based on the clinical picture, supporting findings on chest imaging and successful isolation of the pathogen from CSF cultures in the context of disseminated disease (Figure 5). These observations mirror the pathognomonic findings associated with pulmonary nocardiosis described in the literature [4].

Pulmonary involvement in nocardiosis presents heterogeneously and can range from focal or multifocal nodular lesions to consolidation and cavitation [4]. In a case series of 30 patients with pulmonary nocardiosis, 31% presented with consolidation, 21% had a nodule, 14% had multiple nodules, 10% had pleural effusions, and 3% had multifocal consolidations with multiple nodules [6].

Other potential primary sites of infection are the skin and soft tissues. Cutaneous involvement has been described in only eight percent of cases and ranges from superficial disease (cellulitis, pustules) to mycetomas and lymphocutaneous involvement mimicking sporotrichosis [7]. Infection from direct transcutaneous inoculation is more common than secondary skin involvement due to dissemination [5]. Although our patient did not show evidence of cutaneous involvement on admission, he did endorse having a necrotic lesion in the fifth digit of his left hand around the time of onset of his ocular symptoms three months prior. The lesion resolved on its own, and no tissue was obtained.

Endophthalmitis from Nocardia is rare and usually exogenous in nature in the context of trauma or eye surgery [4]. Endogenous involvement is even less common and tends to occur in immunocompromised individuals as a result of hematogenous spread from a pulmonary source, as suspected in this patient with no history of ocular trauma or surgery [1]. Clinically, slit lamp examination may disclose corneal endothelial exudates, hypopyon, or nodular deposits at the pupillary border. These findings may be associated with minimal vitritis or posterior segment findings. However, progressive disease can present with white fluffy exudates on the posterior capsule and anterior vitreous, which can be mistaken for fungal endophthalmitis as was the case with our patient (Figure 2) [8]. On fundoscopy, it may present as a single white sub-retinal lesion with surrounding hemorrhage and retinal thickening with overlying vitritis (Figure 2) [4]. The classic advanced disease presents as an area of yellowish chorioretinitis with surrounding hemorrhage that can also be associated with sub-retinal abscess formation, optic nerve involvement, or retinal detachment [9]. Traditionally, the treatment for suspected fungal endophthalmitis is intravitreal amikacin with or without pars plana vitrectomy [8].

CNS involvement from Nocardia is more common and has been reported in up to 44% of cases with disseminated disease [3]. Nocardia species are known to have a predisposition to invade and replicate within the CNS. Experiments in mice have shown that several strains of Nocardia have surface receptors that recognize and bind sites on capillary endothelial cells in the CNS. The progression and clearance of the infection depend on the virulence as well as the inoculum of the species invading the brain [2]. N. farcinica has shown to be particularly virulent and has higher rates of CNS penetrance than other subspecies of the same genus [10]. As seen in this patient, the most common predisposing factor associated with CNS invasion is the use of systemic corticosteroids. Imaging features of CNS Nocardiosis are often misdiagnosed as tumors or abscesses, with most cases requiring neurosurgical evacuation to obtain cultures for definitive diagnosis and treatment [2]. An invasive diagnostic approach was not pursued in this case due to the successful isolation of the pathogen from CSF cultures and clinical improvement on current interventions.

Regarding treatment and prognosis, CNS involvement is associated with high morbidity and mortality due to critical delays in treatment secondary to misdiagnosis [1]. Sulfonamides are the most commonly used antibiotics, and drug susceptibilities are important to identify resistant strains [4]. Combination therapy consisting of TMP-SMX and imipenem is considered standard of care for CNS disease [11]. Improved outcomes have been reported with the combined use of sulfonamides for more than six months and neurosurgery [2, 12].

Our patient had a favorable outcome on pharmacological therapy alone. Given his remarkable clinical improvement on noninvasive treatment measures and out of patient preference, a neurosurgical intervention was not attempted.

## Conclusions

CNS nocardiosis is an often fatal disease usually seen in the context of immunodeficiency. Common associations are chronic use of glucocorticoids and organ transplantation. Delay in diagnosis is usually a consequence of nonspecific clinical presentation, nonspecific findings on neuroimaging and variable growth of Nocardia colonies in vitro. The mainstay of treatment remains neurosurgical evacuation and antimicrobial therapy consisting of TMP-SMX and the use of imipenem with or without amikacin. This case underscores the importance of early recognition and treatment to prevent irrevocable neurological compromise stemming from delayed diagnosis.
